# Supplementary material for: A novel bicyclic 2,4-diaminopyrimidine inhibitor of Streptococcus suis dihydrofolate reductase
Source: PeerJ. 2021 Feb 3;9:e10743. doi: 10.7717/peerj.10743 (PMC7866885; doi:10.7717/peerj.10743)
Supplement: Figure S1 — (A) Nucleotide sequence of Ssdhfr gene (Genbank accession number MH388486 and MH388487) and (B) amino acid sequence of Ss DHFR from S. suis P1/7 and S. suis HE06, and (C) amino acid sequence of Ss DHFR and S. pneumoniae DHFR (Sp DHFR) were aligned using Clustal Omega. (Sievers & Higgins, 2014). Amino acids labeled in red are predicted to be folate binding site whereas amino acids labeled in blue are predicted to be NADPH binding site by NCBI conserved domain database. [file peerj-09-10743-s002.pdf]

A.

```
SsP17DHFR      ATGACTAAAAAGATTGTTGCCATTTGGGCTCAAGATGAAAATGGATTGATTGGAAAAGGT  60
SsHE06DHFR     ATGACTAAAAAGATTGTTGCCATTTGGGCTCAAGATGAAAATGGATTGATTGGAAAAGGT  60
               ************************************************************

SsP17DHFR      GATAGACTTCCTTGGTCATTGCCAGCTGACTTAGCTCATTTCAAAAAAACGACAACAGGT  120
SsHE06DHFR     GATAGACTTCCTTGGTCATTGCCAGCTGACTTAGCTCATTTCAAAAAAACGACAACAGGT  120
               ************************************************************

SsP17DHFR      CACACGATGGTGATGGGACGTATAACTTTCGACGGCATGGGTAAACGTGCTCTTCCCAAT  180
SsHE06DHFR     CACACGATGGTGATGGGACGTATAACTTTCGACGGCATGGGTAAACGTGCTCTTCCCAAT  180
               ************************************************************

SsP17DHFR      CGTCACACCATCGTTTTGACAACGGATAAAACCTATCAACTAGAGAGTGAACGTATTACC  240
SsHE06DHFR     CGTCACACCATCGTTTTGACAACGGATAAAACCTATCAACTAGAGAGTGAACGTATTACC  240
               ************************************************************

SsP17DHFR      ATTTTACACAGTGTGGAAGATGTTCTAGATTGGTATAACAAACAGGAGCAGACACTATTT  300
SsHE06DHFR     ATTTTACACAGTGTGGAAGATGTTCTAGATTGGTATAACAAACAGGAGCAGACACTATTT  300
               ************************************************************

SsP17DHFR      GTGATTGGTGGGGGACAAATTTTTACTGCTTTTGCACCTTACATTGAAACATTAATAGTG  360
SsHE06DHFR     GTGATTGGTGGGGGACAAATTTTTACTGCTTTTGCACCTTACATTGAAACATTAATAGTG  360
               ************************************************************

SsP17DHFR      ACAGATATTCACGGTCAATTTAATGGCGATGTGTATTTTCCGAAAGCATTTCCTATGGAG  420
SsHE06DHFR     ACAGATATTCACGGTCAATTTAATGGCGATGTGTATTTTCCGAAAGCATTTCCTATGGAG  420
               ************************************************************

SsP17DHFR      AAATTTCAGCTACAAAGCGCCAATTTACGTCCAAAAGACGAGAAAAACCCGTATGATTTT  480
SsHE06DHFR     AAATTTCAGCTACAAAGCGCCAATTTACGTCCAAAAGACGAGAAAAACCCGTATGATTTT  480
               ************************************************************

SsP17DHFR      ACAATAAAAACCTATGAAAGAAGAGATGGTTAG   513
SsHE06DHFR     ACAATAAAAACCTATGAAAGAAGAGATGGTTAG   513
               *********************************
```

B.

Folate binding - red
NADPH binding - blue

```
SsP17DHFR      MTKKIVAIWAQDENGLIGKGDRLPWSLPADLAHFKKTTTGHTMVMGRITFDGMGKRALPN   60
SsHE06DHFR     MTKKIVAIWAQDENGLIGKGDRLPWSLPADLAHFKKTTTGHTMVMGRITFDGMGKRALPN   60
               ************************************************************

SsP17DHFR      RHTIVLTTDKTYQLESERITILHSVEDVLDWYNKQEQTLFVIGGGQIFTAFAPYIETLIV   120
SsHE06DHFR     RHTIVLTTDKTYQLESERITILHSVEDVLDWYNKQEQTLFVIGGGQIFTAFAPYIETLIV   120
               ************************************************************

SsP17DHFR      TDIHGQFNGDVYFPKAFPMEKFQLQSANLRPKDEKNPYDFTIKTYERRDG             170
SsHE06DHFR     TDIHGQFNGDVYFPKAFPMEKFQLQSANLRPKDEKNPYDFTIKTYERRDG             170
               **************************************************
```

C.

Folate binding - red
NADPH binding - blue

```
SsDHFR      MTKKIVAIWAQDENGLIGKGDRLPWSLPADLAHFKKTTTGHTMVMGRITFDGMGKRALPN    60
SpDHFR      MTKKIVAIWAQDEEGVIGKDNRLPWHLPAELQHFKETTLNHAILMGRVTFDGMGRRLLPK    60
            *************:*:***.:**** ***:* ***:** .*:::***:******:* **:

SsDHFR      RHTIVLTTDKTYQLESERITILHSVEDVLDWYNKQEQTLFVIGGGQIFTAFAPYIETLIV    120
SpDHFR      RETLILTRNPEEKI--DGVATFQDVQSVLDWYQAQEKNLYIIGGKQIFQAFEPYLDEVIV    118
            *.*::** :   ::  : :: ::.*:.*****: **:.*::*** *** ** **:: :**

SsDHFR      TDIHGQFNGDVYFPKAFPMEKFQLQSANLRPKDEKNPYDFTIKTYERRDG              170
SpDHFR      THIHARVEGDTYFPEELDLSLFETVSSKFYAKDEKNPYDFTIQYRKRKEV              168
            *.**.:.:**.***: : :. *:  *:::  ***********:  :*::
```
